# PINK1 mediated mitophagy attenuates early apoptosis of gingival epithelial cells induced by high glucose

**DOI:** 10.1186/s12903-022-02167-5

**Published:** 2022-04-26

**Authors:** Chunhui Zhu, Ying Zhao, Dandan Pei, Zhongbo Liu, Jin Liu, Ye Li, Shuchen Yu, Lingyan Ma, Junyi Sun, Ang Li

**Affiliations:** 1 Key Laboratory of Shaanxi Province for Craniofacial Precision Medicine Research, College of Stomatology, Xi’an Jiaotong University, No. 98, Xiwu Road, Xincheng District, Xi’an, 710004 China; 2 Department of Periodontology, College of Stomatology, Xi’an Jiaotong University, Xi’an, China; 3 Department of Special Clinic, College of Stomatology, Xi’an Jiaotong University, Xi’an, China

**Keywords:** PINK1, Mitophagy, Apoptosis, Short-term high glucose, Human gingival epithelial cells, Diabetes mellitus, Periodontitis

## Abstract

**Background:**

Oxidative stress mediated by hyperglycemia damages cell-reparative processes such as mitophagy. Down-regulation of mitophagy is considered to be a susceptible factor for diabetes mellitus (DM) and its complications. However, the role of mitophagy in DM-associated periodontitis has not been fully elucidated. Apoptosis of human gingival epithelial cells (hGECs) is one of the representative events of DM-associated periodontitis. Thus, this study aimed to investigate PTEN-induced putative kinase 1 (PINK1)-mediated mitophagy activated in the process of high glucose (HG)-induced hGECs apoptosis.

**Methods:**

For dose–response studies, hGECs were incubated in different concentrations of glucose (5.5, 15, 25, and 50 mmol/L) for 48 h. Then, hGECs were challenged with 25 mmol/L glucose for 12 h and 48 h, respectively. Apoptosis was detected by TdT-mediated dUTP nick end labeling (TUNEL), caspase 9 and mitochondrial membrane potential (MMP). Subsequently, autophagy was evaluated by estimating P62, LC3 II mRNA levels, LC3 fluorescent puncta and LC3-II/I ratio. Meanwhile, the involvement of PINK1-mediated mitophagy was assessed by qRT-PCR, western blotting and immunofluorescence. Finally, hGECs were transfected with shPINK1 and analyzed by MMP, caspase 9 and annexin V-FITC apoptosis.

**Results:**

The number of TUNEL-positive cells and caspase 9 protein were significantly increased in cells challenged with HG (25 mmol/L) for 48 h (HG 48 h). MMP was impaired both at HG 12 h and HG 48 h, but the degree of depolarization was more serious at HG 48 h. The autophagy improved as the amount of LC3 II increased and p62 decreased in HG 12 h. During this process, HG 12 h treatment induced PINK1-mediated mitophagy. PINK1 silencing with HG 12 h resulted in MMP depolarization and cell apoptosis.

**Conclusions:**

These results suggested that loss of the PINK1 gene may cause mitochondrial dysfunction and increase sensitivity to HG-induced apoptosis of hGECs at the early stage. PINK1 mediated mitophagy attenuates early apoptosis of gingival epithelial cells induced by high glucose.

**Supplementary Information:**

The online version contains supplementary material available at 10.1186/s12903-022-02167-5.

## Background

Diabetes mellitus (DM) has emerged as a global health challenge. According to statistics from the international diabetes federation, it is estimated that one person dies of diabetes or its complications every seven seconds [1]. DM is characterized by hyperglycemia resulting from inadequate insulin production, and/or impaired insulin action. It is well documented that hyperglycemia, especially when poorly controlled, is a major independent factor in the onset, progression and aggravation of periodontitis [2]. In addition, in the early stages of diabetes, the host may promote tissue repair to resist the damage of hyperglycemia. These facts suggest the importance early treatment of periodontitis in subjects with DM.

Periodontitis is a localized infectious disease of tooth-supporting tissues caused by bacteria, such as *Porphyromonas gingivalis* [3]. Bacterial products influenced the gingival tissues, activating cellular processes that induce the destruction of the connective tissue and alveolar bone [4]. Gingival epithelial cells (GECs) are the first line of defense in periodontal tissue against oral bacterial and other harmful stimuli, such as high blood glucose and smoking [5]. Periodontal bacteria and virulence factors increase the number of apoptotic GECs in the gingival epithelium of patients with periodontitis [6]. Compared with periodontitis in non-DM, the percentage of apoptotic GECs and the degree of inflammatory cells infiltration were increased significantly in rats with DM-associated periodontitis (DMPD) [7]. The apoptosis of human GECs triggers the destruction of epithelial barrier and aggravated the progression of DMPD. Taken all the factors into consideration, it is suggested that apoptosis of GECs is one of the signs of DMPD, which can be used to predict the pathological progress of this disease. Therefore, it is reasonable to assume that intervention of pathologic apoptosis of GECs would alleviate periodontitis under diabetic condition.

Apoptosis is an important pathway for cell death, and mitochondria-dependent apoptosis is a special kind. Mitochondria play a central role in cellular energy metabolism [8]. When the cells are subjected to external stimuli, they activate cysteinyl aspartate specific proteinase (Caspase), which eventually leads to apoptosis and DNA damage of GECs in the progression of periodontitis. Caspase activation is a classic apoptotic pathway characterized by decreased mitochondrial membrane potential (MMP), increased permeability and activated caspase apoptosis protein [9]. Mitophagy, a selective type of autophagy, is a major mechanism to eliminate damaged mitochondria and stabilize mitochondrial function [10]. PTEN-induced putative kinase 1 (PINK1) / Parkin, Bcl 2 / adenovirus E1B 19 kDa protein-interacting protein 3 (BNIP3) / Nip-like protein X (NIX), and Fun 14 Domain containing 1 (FUNDC1) are three crucial receptor proteins of mitophagy [11]. Growing evidence indicates that disruption of mitophagy can lead to mitochondrial dysfunction and apoptosis, acerbating diabetes-related complications [12, 13]. Mutations at the PINK1 locus have also been reported to be susceptible to type 2 DM [14]. Although periodontitis is considered the sixth major complication of diabetes [2], the involvement and possible molecular mechanisms of mitophagy in the context of DMPD have not been fully understood. Herein, we aimed to investigate the role of mitophagy in pathologic apoptosis of GECs under hyperglycemic conditions and thus the underlying molecular mechanisms.

The pathological changes in GECs resulting from high glucose (HG) have been widely accepted in the DM model [15]. In this study, human GECs (hGECs) were employed to induce apoptosis using glucose under different concentrations (5.5, 15, 25, and 50 mmol/L) and stimulating timings (12 h and 48 h). Apoptosis was detected by TdT-mediated dUTP nick end labeling (TUNEL), caspase 9 and MMP. Then, the autophagy induced by HG at the indicated concentration (25 mmol/L) and timings (12 h and 48 h) was identified by LC3 II and P62. hGECs in HG conditions were further used to assess the mRNA change of mitophagy including *FUNDC1*, *NIX*, and *PINK1*. Finally, the effects of downregulated *PINK1*-mediated mitophagy on apoptosis were investigated by MMP, caspase 9 and annexin V-FITC apoptosis. This study aimed to investigate the role of PINK1-mediated mitophagy in HG-triggered apoptosis at the early stage.

## Methods

### Study design

HGECs were stimulated by glucose at different times to induce apoptosis. For dose–response studies, hGECs were incubated in different concentrations of glucose (5.5, 15, 25, and 50 mmol/L) for 48 h. TUNEL, caspase 9 and MMP were detected. We determined that the lowest concentration of HG-induced apoptosis of hGECs was 25 mmol/L. Then, hGECs were challenged with 25 mmol/L glucose for 12 h and 48 h, respectively. Cells were divided into the following groups: normal glucose (NG) 12 h group (cells were cultured for 12 h with 5.5 mmol/L glucose); HG 12 h group (cells were cultured for 12 h with 25 mmol/L glucose); NG 48 h group (cells were cultured for 48 h with 5.5 mmol/L glucose); HG 48 h group (cells were cultured for 48 h with 25 mmol/L glucose). Then, the autophagy under different conditions was assessed by LC3 II and P62. In addition, quantitative real-time polymerase chain reaction (qRT-PCR), western blotting and immunofluorescence staining were performed to evaluate mitophagy. hGECs were transfected with shPINK1 and analyzed by MMP, caspase 9 and annexin V-FITC apoptosis.

### Cell culture

The primary hGECs (HUM-iCell-m016) was commercial used and purchased from the company (Cybertron Biotechnology Co., Ltd., Shanghai, China). Human gingival tissues were obtained with Institutional Review Board approval from healthy patients after third molar extraction. This study was approved by the Ethical Committee, Faculty of Medicine, Xi’an Jiaotong University (No. 2019-952) and strictly followed the ethical guidelines of the declaration of Helsinki of the World Medical Association. The procedure was fully explained to the patients and all the donors signed informed consent. hGECs were cultured using the direct explant method as previously described [16]. Briefly, after disinfecting in povidone iodine solution and rinsing in phosphate-buffered saline with 1% antibiotics. The gingival tissues were treated with 0.025% trypsin and 0.01% EDTA overnight at 4 °C, around 18 hours. Afterward the trypsin reaction was stopped using DMEM and the epithelial layer was carefully separated from the connective tissue. the epithelial layer samples were cut into pieces approximately 1 mm^3^ in size. Then, the cell suspension was centrifuged at 1000 r/min for 5 min, and the pellet was suspended in K-SFM medium (Invitrogen Life Technologies) containing 10 μg/ml insulin, 5 μg/ml transferrin, 10 μM 2-ME, 10 μM 2-aminoethanol, 10 mM sodium selenite, 50 μg/ml bovine pituitary extract, 100 U/ml penicillin/streptomycin, and 50 ng/ml fungizone (complete medium). When the squamous-shaped epithelial cells began to expand around the gingival tissue sample to a diameter of 2–5 mm, the tissue was removed. Cells were cultured with K-SFM medium in a 37°C incubator with 5% CO_2_. The culture medium was changed every 2–3 days. When the cells grew to 80–90%, they were harvested and subcultured.

### TUNEL assay

Apoptotic cells were detected by a TUNEL detection kit (KeyGen Biotechnology Co., Ltd, Jiangsu, China) according to the manufacturer’s protocol [17]. Briefly, cells were fixed with 4% paraformaldehyde for 30 min and permeabilized with 1% Triton X-100 for 5 min at room temperature. Cells were cultured in 50 μL TdT enzyme reaction solution for 1 h at 37 °C. After washing with phosphate-buffered saline, cells were incubated in 50μL of Streptavidin-TRITC working solution at 37 °C for 30 min. The nuclei were counterstained with 4′, 6-diamidino-2-phenylindole (DAPI) at room temperature for 10 min. The whole experiment was carried out in the dark. TUNEL-positive cells were observed and photographed using a laser scanning confocal microscope (LSCM; Olympus Fluoview FV3000, Olympus Corporation, Tokyo, Japan) at excitation/emission wavelengths of 470/550 nm. Five representative fields were selected from each group and the TUNEL-positive cells in each field were calculated by ImageJ software (National Institutes of Health, Bethesda, MD, USA).

### Western blotting

Total protein from hGECs treated with different concentrations of glucose was extracted using RIPA lysis buffer with 1% phosphatase inhibitor and 1% PMSF. Then, the protein concentration was measured by the BCA Protein Assay Kit (BOSTER Biological technology Co., Ltd, Wuhan, China) according to the manufacturer’s instructions. Protein expression levels were detected through western blotting as described previously [18]. The following primary antibodies were used: caspase 9 (1:1000 dilution; Cell Signaling Technology Inc., Danvers, MA, USA), LC3 (1:1000 dilution; Cell Signaling Technology Inc., Danvers, MA, USA), PINK1 (1:1000 dilution; Abcam Inc., Cambridge, Massachusetts, USA), and glyceraldehyde-3-phosphate dehydrogenase (GAPDH) (1:2000 dilution; Bioss Biotechnology Co., Ltd, Beijing, China). Horseradish peroxidase conjugated goat anti-rabbit IgG (1:2000 dilution; BOSTER Biological technology Co., Ltd, Wuhan, China) was used as a secondary antibody. The protein signals were detected by electrogenerated chemiluminescence substrates. The protein gel imaging analysis system (Syngene, United Kingdom) was used to detect grayscale values of bands.

### MMP

MMP was measured by a 5,5′,6,6′-tetrachloro-1,1′,3,3′-tetraethylbenzimidazolcarbocyanine iodide (JC-1) kit (Beyotime, Beyotime Biotechnology Co., Ltd., Shanghai, China) according to the method described previously [19]. hGECs were seeded in laser confocal dishes at a density of 8 × 10^4^ cells per well. Cells were incubated in 1 mL JC-1 (10 μm) staining solution for 30 min at 37 °C in the dark. Then, the cells were washed twice with JC-1 staining buffer. Cells were observed under LSCM at 530 nm wavelengths for green fluorescence and 590 nm wavelengths for red fluorescence. The ratio of red/green fluorescence intensity was used to measure the depolarization of MMP.

### QRT-PCR

Total RNA was isolated from hGECs using TRIzol™ reagent (Invitrogen Corp., Carlsbad, CA) following the manufacturer’s instructions as described previously [20]. RNA concentration was determined with a NanoDrop 2000c spectrophotometer (Thermo Fisher Scientific Inc., Waltham, MA, USA). Precipitated RNA was dissolved in diethylpyrocarbonate water and reverse transcribed into cDNA using PrimeScript™ RT Master Mix (TaKaRa Biotechnology Co., Ltd., Dalian, China). Primer sequences used for qRT-PCR analysis (*LC3 II* forward primer 5′-AGCAGCATCCAACCAAAA-3′, reverse primer 5′-CTGTGTCCGTTCACCAACAG-3′; *P62* forward primer 5′- TGCCCAGACTACGACTTGTG-3′, reverse primer 5′-GAGAAGCCCTCAGACAGGTG-3′; *PINK1* forward primer 5′-GGACGCTGTTCCTCGTTA-3′, reverse primer 5′-ATCTGCGATCACCAGCCA-3′; *NIX* forward primer 5′-AGTAGCTTATTTGAACTTGAGACCATTG-3′, reverse primer 5′-TGAGGGTTACTGGAATTGGATATGTA-3′; *FUNDC1* forward primer 5′-GCAGTAGGTGGTGGCTTTC-3′, reverse primer 5′-TGCTTTGTTCGCTCGTTT; *GAPDH* forward primer 5′-GCACCGTCAAGGCTGAGAAC-3′, reverse primer 5′-TGGTGAAGACGCCAGTGGA-3′). qRT-PCR was performed on the Applied Biosystems 7500 Fast Real-Time PCR System with SYBR® Premix Ex Taq™ II (TaKaRa Biotechnology Co., Ltd., Dalian, China) according to the manufacturer’s protocol. The obtained CT values were calculated by the 2^−ΔΔCt^ method. The results of relative gene levels were normalized to GAPDH.

### Immunofluorescence

The cellular localization of LC3 II and PINK1 was examined by LSCM as described previously [21]. Briefly, hGECs were seeded in laser confocal dishes at a density of 8 × 10^4^ cells per well. After treatment, cells were fixed for 20 min with 4% precooled paraformaldehyde and washed with phosphate-buffered saline. Then, the cells were permeabilized with 0.2% Triton X-100 for 10 min and blocked with 5% BSA for 30 min at room temperature. hGECs were fixed and incubated with LC3 II (1:100 dilution; Cell Signaling Technology Inc., Danvers, MA, USA) or PINK1 (1:100 dilution; Abcam Inc., Cambridge, Massachusetts, USA), followed by FITC-labeled goat anti-rabbit IgG fluorescent secondary antibody (1:1000 dilution; GenePharma Co., Ltd., Shanghai, China). Then, the cells were counterstained with DAPI (1:1000 dilution; Sigma-Aldrich Trading Co. Ltd., St. Louis, MO, USA) for visualization of the nuclei. Cell images were further observed and photographed using LSCM at excitation/emission wavelengths of 488/530 nm. Fluorescence intensity was calculated using ImageJ software.

### Lentivirus transfection of PINK1

PINK1 inhibition by lentivirus-mediated shRNA vector and its corresponding empty vectors were designed and synthesized by GenePharma (GenePharma Co., Ltd., Shanghai, China). hGECs were seeded on six-well plates and allowed to attach overnight. At approximately 60–70% confluence, hGECs were transfected with shPINK1 or shNC. The virus was added with 5 μg/mL polybrene and incubated with cells in 5% CO_2_ at 37 °C. After 18 h, the supernatant fluid with the viral particles was removed and replaced with fresh medium. Then, the cells were incubated in 5% CO_2_ at 37 °C for an additional 72 h. The fluorescence efficiency of the cells was observed by fluorescence microscopy.

### Flow cytometric analysis

Apoptosis in hGECs was assessed by an annexin V-FITC/PI apoptosis analysis kit (KeyGen, KeyGen Biotechnology Co., Ltd., Nanjing, China) according to the manufacturer’s protocol [22]. Briefly, cells were digested with 500 μL trypsin without EDTA and centrifuged for 5 min at 2000 rpm. Then, the cells were resuspended in 500 μL binding buffer stained with 5 μL FITC-conjugated annexin V and 5 μL of propidium iodide for 15 min at room temperature in the dark. Quantitative analysis of apoptosis was detected by flow cytometry (Becton, Dickinson and Company, USA) in 1 h at excitation/emission wavelengths of 488/530 nm. The percentage of apoptotic events in a given area was counted as the percentage of total apoptotic cells.

### Statistical analysis

All experiments were performed at least in triplicate. Quantitative analysis was expressed as the mean ± standard deviation (SD). One-way analysis of variance and post hoc tests were further compared for each group using SPSS 22.0. The data were considered significant at *P* < 0.05.

## Results

### Establishment of a stable model of HG-induced apoptosis of hGECs

**Fig. 1:**
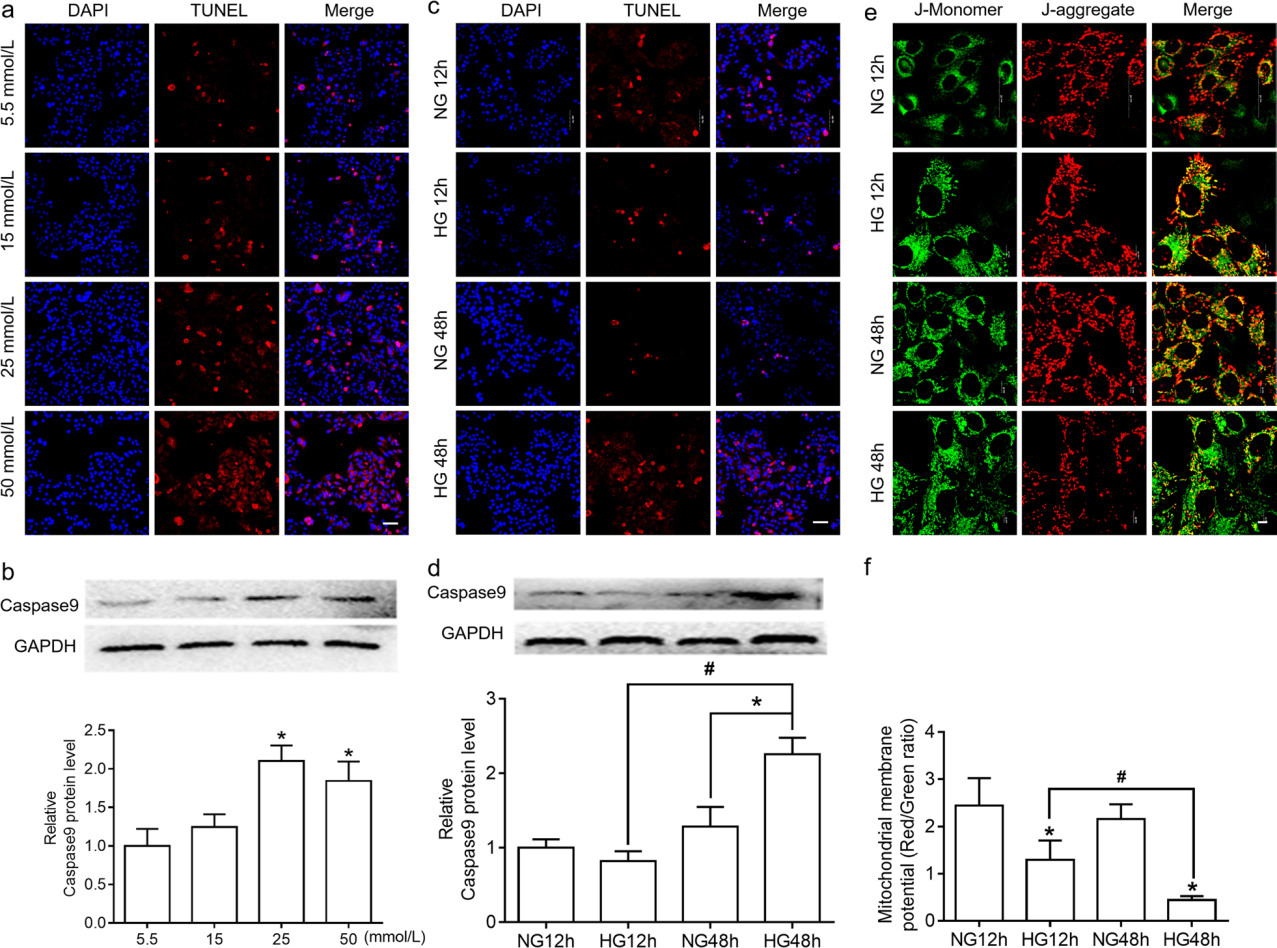
The effect of HG on apoptosis in hGECs. **a** hGECs were treated with 5.5, 15, 25, and 50 mmol/L glucose for 48 h. Apoptotic cells were identified by TUNEL assay. Scale bar = 80 μm. **b.** The protein level of caspase 9 was detected by western blotting (representative images and statistical analysis). *P < 0.05 versus 5.5 mmol/L. ^#^*P* < 0.05. **c.** hGECs were treated with or without 25 mmol/L glucose for 12 and 48 h. Apoptotic cells were identified by TUNEL assay. Scale bar indicates 80 μm. **d.** The protein level of caspase 9 was detected by western blotting (representative images and statistical analysis). *P < 0.05 versus NG 12 h. ^#^*P* < 0.05. **e.** MMP was measured by LSCM analysis of JC-1 staining. Scale bar = 10 μm. **f.** The ratio of red/green fluorescence intensity was assembled from the statistical analysis. Significant differences are shown as *P < 0.05 versus NG 12 h. ^#^*P* < 0.05. Data from representative experiments are shown as the mean ± SD from a minimum of triplicates per experiment. The original picture of western blots of Fig. 1b, 1d were shown in Additional file 1: Fig. S1b, S1d

We intended to determine the role of HG in apoptosis of hGECs. hGECs were first challenged with different concentrations of glucose for 48 h. As shown in Fig. 1a, compared to the normal glucose group (5.5 mmol/L glucose), the apoptotic cells presented red fluorescence in a dose-dependent manner. In addition, 25 and 50 mmol/L glucose treatments stimulated caspase 9 protein cleavage (Fig. 1b). These results suggested that 25 mmol/L was the lowest concentration of HG to induce apoptosis of hGECs.

Apoptosis was further examined by challenges with 25 mmol/L glucose for 12 h and 48 h. Treatment with 25 mmol/L glucose for 48 h significantly increased the percentage of apoptotic cells when compared to that of the NG 48 h group. The percentage of TUNEL-positive cells at 48 h was significantly higher than that of 12 h (Fig. 1c). Similarly, caspase 9 expression exhibited the same trend as TUNEL (Fig. 1d). Therefore, these results indicated that notable hGECs apoptosis was induced using 25 mmol/L glucose for 48 h.

MMP is an indicator of mitochondrial health and an important parameter of mitochondrial function. Changes in cell MMP can be directly detected by the fluorescent probe JC-1. In the mitochondria, JC-1 presented a potential dependent accumulation in the mitochondria, which showed a fluorescence shift from green (emission wavelength: ~ 529 nm) to red (emission wavelength: ~ 590 nm). JC-1 can be optically measured by the red/green fluorescence intensity ratio. We examined whether the depolarization of MMP was involved in HG-induced hGECs apoptosis. As shown in Fig. 1e, the MMP in the NG 12 h and NG 48 h groups was maintained at a normal level with red fluorescence. HG stimulated a loss of MMP both at 12 h and 48 h with increased green fluorescence. In addition, compared to the HG 12 h group, the red/green fluorescence intensity ratio was significantly lower in HG 48 h (Fig. 1f). According to the result, we found that MMP depolarization occurred as early as 12 h after stimulation by HG.

### Effect of HG on autophagy in hGECs

**Fig. 2:**
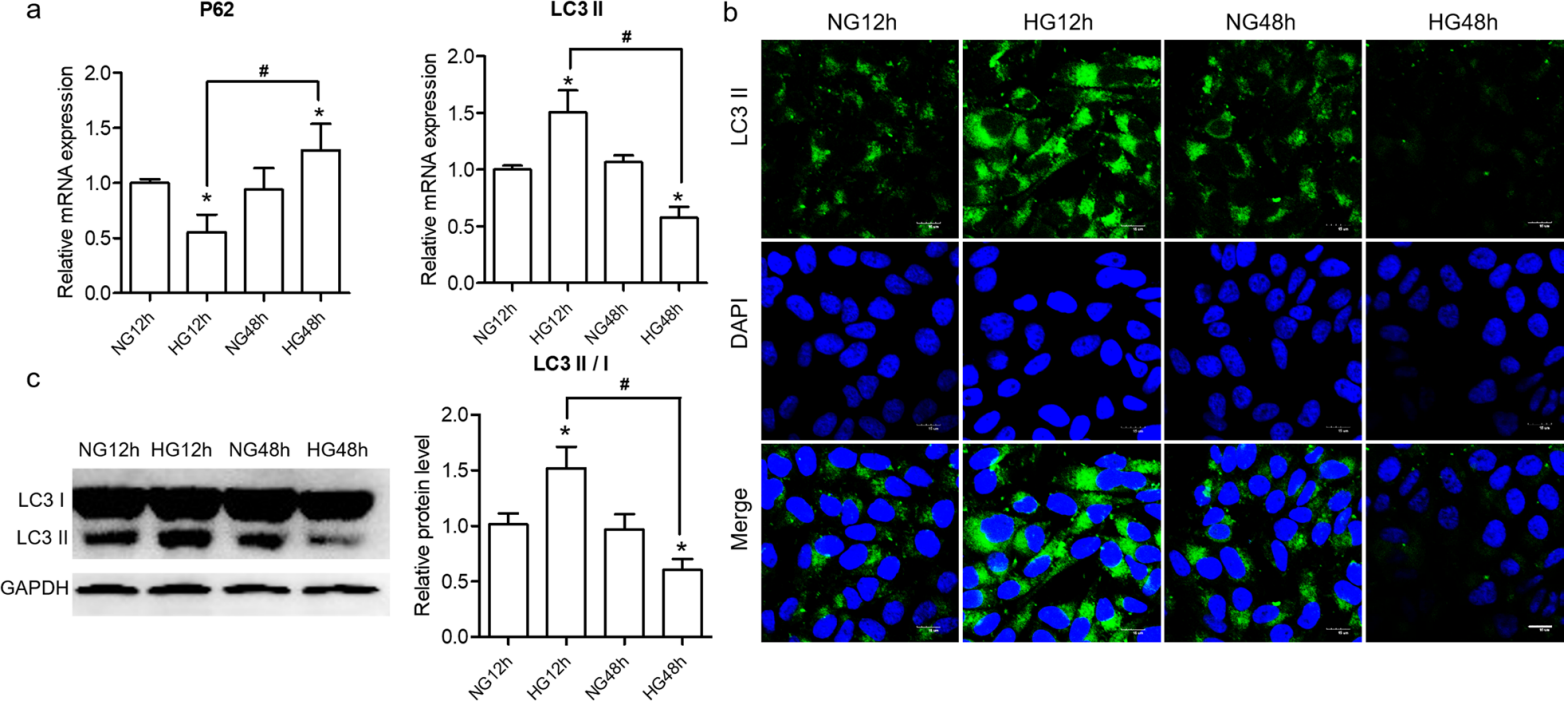
The effect of HG on autophagy. **a** The relative *LC3 II* and *P62* mRNA was measured by qRT-PCR. LC3 II was measured by LSCM (**b**) and western blotting (**c**) analysis. Scale bar = 15 μm. The ratio of LC3 II/LC3 I was assembled from the statistical analysis. Significant differences are shown as *P < 0.05 versus NG 12 h. ^#^*P* < 0.05. The original picture of western blot of Fig. 2c was shown in Additional file 1: Fig. S2c

Next, we examined LC3 II and P62 to further confirm the effect of HG on autophagy. As shown in Fig. 2a, HG 48 h treatment significantly increased the expression of *P62* mRNA in the hGECs when compared to that of NG 12 h and HG 12 h. Moreover, the expression of *LC3 II* mRNA was significantly elevated in the HG 12 h group compared with mRNA level in NG 12 h or HG 48 h. We also monitored autophagic activity by LC3 using immunofluorescence and western blotting analysis. Immunofluorescence data presented in Fig. 2b, LC3 II in the NG 12 h and NG 48 h groups was maintained at a normal level with green fluorescence. HG stimulated strong fluorescence intensity at 12 h and weak fluorescence intensity at 48 h. Western blotting assay further confirmed that the relative ratio of LC3 II/I significant increase in HG 12 h (Fig. 2c). In conclusion, the results from Fig. 2 indicated autophagy occurred in HG 12 h with the evidence of down-regulated p62 and increased LC3 levels.

### Effect of HG on PINK1-mediated mitophagy in hGECs

**Fig. 3:**
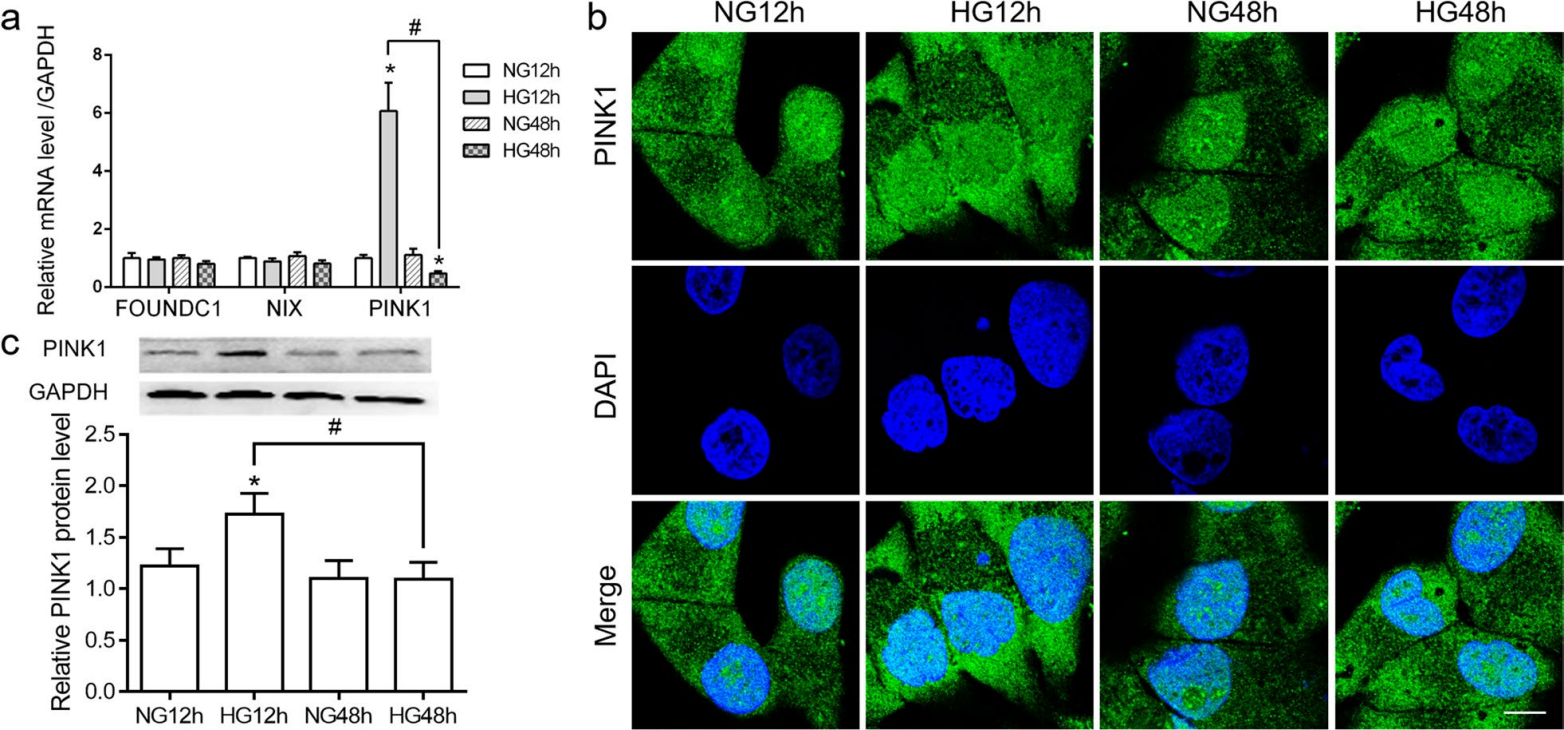
The effect of high glucose HG on mitophagy. HGECs were challenged with or without 25 mmol/L glucose for 12 h and 48 h. **a** *FUNDC1*, *NIX*, and *PINK1* mRNA levels were determined by qRT-PCR. **b** The fluorescence of PINK1 was detected by LSCM. **c.** The protein level of PINK1 was measured by western blotting (representative images and statistical analysis). Significant differences are shown as *P < 0.05 versus NG 12 h. ^**#**^*P* < 0.05. The original picture of western blot of Fig. 3c was shown in Additional file 1: Fig. S3c

Mitophagy is an effective process in eukaryotic cells to remove excessive or damaged mitochondria. The mRNA levels of representative genes in the mitophagy pathway (*FUNDC1, NIX, PINK1*) were assessed by qRT-PCR. We found a significant increase in the expression of *PINK1* mRNA in HG 12 h. However, there were no significant differences in the expression of *FUNDC1* and *NIX* mRNA between groups (Fig. 3a). Furthermore, the expression of PINK1 was verified using immunofluorescence and western blotting. Immunofluorescence analysis showed that the relative fluorescence intensity of PINK1 in the HG 12 h group was higher than that in the NG 12 h, NG 48 h, and HG 48 h groups (Fig. 3b). This result was further confirmed by western blotting using a specific antibody against PINK1 (Fig. 3c).

The results from Fig. 3 suggested that PINK1-mediated mitophagy might be compromised by stimulation of HG in hGECs. Moreover, we found that HG triggered PINK1-dependent mitophagy at 12 h.

### Effect of PINK1 silencing on MMP and apoptosis in hGECs

**Fig. 4:**
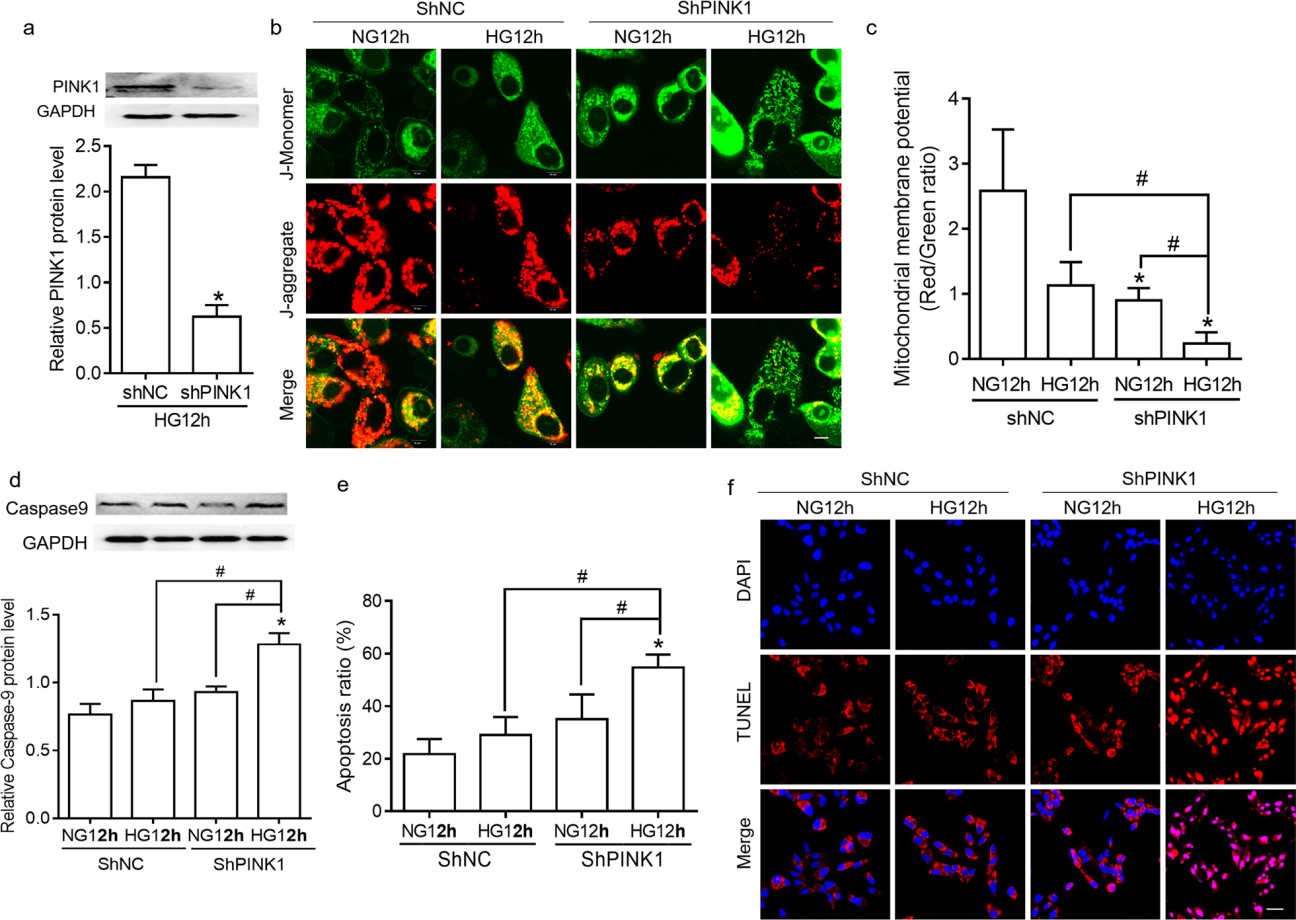
The effect of silencing the *PINK1* gene on mitochondrial dysfunction and apoptosis of hGECs at HG 12 h. HGECs were transfected with ShPINK1 and its corresponding empty vector ShNC with or without 25 mmol/L glucose for 12 h. **a** The protein level of PINK1 was determined by western blotting (representative images and statistical analysis). **b** MMP was measured by LSCM analysis of JC-1 staining. Scale bar = 10 μm. **c** The ratio of red/green fluorescence intensity was assembled from the statistical analysis. **d** The protein level of caspase 9 was analyzed by western blotting (representative images and statistical analysis).** e** Apoptotic cells were measured using annexin V/PI by fluorescence-activated cell sorting. The percentage of apoptotic events was assembled from the statistical analysis. **f** Apoptotic cells were identified by TUNEL assay. Scale bar = 80 μm. Significant differences are shown as *P < 0.05 versus NG 12 h-ShNC. ^#^*P* < 0.05. The original picture of western blots of Fig. 4a, 4d were shown in Additional file 1: Fig. S4a, S4d

The ShRNA lentiviral vector with *PINK1* gene silencing was constructed and transfected into hGECs. As shown in Fig. 4a, PINK1 protein expression in the ShPINK1 group was significantly lower than that of its negative empty vector ShNC. Moreover, ShPINK1 caused a loss of MMP both in the NG 12 h-ShPINK1 group and HG 12 h-ShPINK1 group, as shown by the loss of the red signal and increased green signal (Fig. 4b). In addition, the JC-1 red/green fluorescence intensity ratio was decreased in HG 12 h-shPINK1 cells compared with NG 12 h-shPINK1 cells (Fig. 4c).

We further explored the role of PINK1-mediated mitophagy of cells in HG 12 h. As expected, the results showed that caspase 9 expression was elevated in the HG 12 h-ShPINK1 group (Fig. 4d). Furthermore, the percentage of apoptotic cells in the HG 12 h-ShPINK1 group was higher than that of HG 12 h-ShNC and NG 12 h-ShPINK1 (Fig. 4e,f).

## Discussion

Prolonged hyperglycemia in diabetic patients leads to excessive production of ROS, which has been demonstrated to aggravate the inflammation, damage the periodontal tissue or impair the tissue repair process [15, 23]. Hyperglycemia increases the severity of infection in diabetic patients by interfering with neutrophil function [24]. The results showed that patients with periodontitis presented significantly more inflammatory mediators, such as TNF-α, IL-6, IL-8, ADMA, Galectin-3, in comparison with healthy subjects [25–27]. Activation of osteoclasts and collagenase results in greater and faster periodontal destruction [28]. Consequently, the therapeutic approach to reduce or eliminate inflammation or oxidative stress is of great importance to patients with DMPD [29, 30].

The present study demonstrated that with increasing culture time of HG, there is a substancial rise in mitochondrial-dependent apoptosis, as evidenced by increased TNUEL-positive cells, caspase 9 and reduced MMP. Cells in the early stage of HG 12 h exhibited an adaptive increase in PINK1 expression, whereas cells in the later stage of HG 48 h displayed attenuated expression of PINK1. Consistent with our research, Bhansali et al. found that mitophagy-related markers of monocytes in prediabetic patients increased, while the corresponding indicators in patients with advanced diabetes decreased [31]. Our assumption of the important role of mitophagy in HG-induced apoptosis in GECs is verified by the results that downregulate PINK1 in GECs aggravated mitochondrial dysfunction and thus further apoptosis, suggesting the crucial role of PINK1 in the protective role of mitophagy in HG-induced pathologic apoptosis in GECs.

In our pre-experiment, we set up an osmotic control group as 5.5 mmol/L glucose plus with 19.5 mmol/L mannitol for either 12 h or 48 h. The results showed that there was no significant difference in the level of cell proliferation and apoptosis protein expression between the osmotic control group and the HG group, which suggested that the apoptosis induced by HG was not caused by hyperosmolarity (data not show). In addition, Zhang et al. has also reported that 25 mmol/L isotonic stimulation had no effect on apoptosis [32]. Besides its pathologic roles in cardiovascular, retinal, and renal disorders [33–35], hyperglycemia is also considered as one of the most important risk factors in the development of periodontitis [36]. However, it is still difficult to evaluate the effect of hyperglycemia on apoptosis in DMPD due to the discrepancy of previous studies. Human periodontal ligament fibroblasts were cultured in different concentrations of glucose to induce apoptosis in a concentration-dependent manner [37]. In another study, 11 mmol/L and 22 mmol/L glucose suppressed proliferation and increased apoptosis of human periodontal endothelial cells [38]. In our study, 15 mmol/L glucose has no significant effect on apoptosis in hGECs. According to the TUNEL and caspase 9 results, the lowest concentration of glucose to induce apoptosis in hGECs was 25 mmol/L.

Mitochondria are considered pivotal organelles in intrinsic apoptotic pathways. Mitochondrial membrane fission is an important manifestation of mitochondrial damage. The number of bilayer membrane vacuoles in retinal pigment epithelial cells (ARPE-19) increases significantly under high glucose (30 mmol/L). In addition, the fact that the number of autophagosomes, the intracellular reactive oxygen species (ROS) levels, and the expression levels of PARKIN, PINK1, BNIP3L, and LC3-II were significantly increased suggested that mitophagy occurred [39]. In this study, increased LC3II, PINK1 and down-regulated *P62* levels were detected in HG 12 h. Intriguingly, MMP depolarization also occurred at 12 h. It showed that the loss of MMP at 12 h was an early manifestation of apoptosis. After HG stimulation persisted for 48 h, the MMP transition pore was irreversibly opened, which resulted in MMP loss and outer membrane rupture. Finally, the cells entered an irreversible apoptotic state. Since mitochondrial dysfunction precedes morphologic alterations, it would permit an early treatment with DMPD.

Mitophagy is considered an important mechanism that controls mitochondrial quality [40]. There is a growing number of evidence indicating that mitophagy increased in various DM-related complications [12, 13, 41]. PINK1/parkin-induced mitophagy is one of the crucial mechanisms through which autophagy reduces the tendency of cells to undergo apoptosis. Although most studies have focused on the PINK1/parkin pathway under high glucose stimulation, other mitophagy pathways have also been involved [42, 43]. Huang et al. reported that high glucose increased the number of autophagosomes and the expression of BNIP3L to protect against morphological changes and ROS generation in retinal pigment epithelial cells [39]. Our present study demonstrated that high glucose stimulated *PINK1* expression but not *FOUNDC* or *NIX*. These results might indicate that high glucose triggered mitophagy in hGECs cells in a PINK1-dependent manner.

In recent years, it has been reported that mitophagy is closely related to apoptosis, and the relationship between them has attracted widespread attention. Platelets in diabetic patients showed increased ROS production, impaired mitochondria and increased biomarkers of apoptosis. After exposure to the autophagy inhibitor 3-methyladenine, almost all platelets lost MMP and increased annexin V apoptosis. Similar results were obtained in *Parkin-/-* mice [12]. Onphachanh et al. used siRNA to inhibit *PINK1* gene expression in a high glucose microenvironment. The results showed that mitochondrial ROS generation and apoptotic protein expression were significantly up-regulated [44]. Many studies have shown that PINK1-dependent mitophagy plays an anti-apoptotic protective role; however, not all evidence is consistent with this conclusion. Tsuda et al. revealed the pathological role of autophagy in periodontitis, in which inhibition of autophagy with 3-methyladenine attenuated butyrate-induced cell death in the gingival epithelial cell line (Ca9-22) [45]. Obviously, the present study highlighted loss of the *PINK1* gene increase sensitivity of hGECs to apoptosis under short-term high glucose conditions. It should be noted that 25 mmol/L glucose induced an increase in PINK1 at 12 h and a decline at 48 h. The differences in the results may be attributed to the fact that enhanced mitophagy is an early response to promote survival by removing damaged mitochondria. When external stimuli are enhanced and mitophagy is disrupted, pro-apoptotic proteins are excessively released and apoptosis is ultimately activated. Therefore, in the process of HG-induced mitophagy, future research will focus on monitoring the degree of mitophagy, tracking the state of mitophagy, and maintaining mitophagy at an adaptive level (Additional file 1: Figures S1b, S1d, S2c, S3c, S4a, S4d).

## Conclusion

In the present study, we demonstrate that the loss of *PINK1* gene increase sensitivity of hGECs to apoptosis under short-term HG conditions. An “adaptive” increase in mitophagy at HG 12 h may delay progression to apoptosis. Down-regulation of PINK1 could accelerate the apoptosis of hGECs. We speculated that in diabetic patients, low expression of *PINK1* gene is closely related to the high risk of periodontitis. PINK1-mediated mitophagy might provide a new target for rescuing HG-induced apoptosis in hGECs to meet the needs of therapy for DMPD at the early stage.

## Supplementary Information

**Additional file 1.** The original picture of western blots of Figs. 1b, 1d, 2c, 3c, 4a, 4d were shown in Figs. S1b, S1d, S2c, S3c, S4a, S4d.

## Data Availability

The datasets during the current study are available from the first author upon request. Contact e-mail: dentistzch@163.com.

## Abbreviations

DM: Diabetes mellitus
GECs: Gingival epithelial cells
DMPD: Diabetes mellitus-associated periodontitis
MMP: Mitochondrial membrane potential
PINK1: PTEN-induced putative kinase 1
BNIP3: Bcl2/adenovirus E1B 19 kDa protein-interacting protein 3
NIX: Nip-like protein X
FUNDC1: Fun14 Domain containing 1
HG: High glucose
qRT-PCR: Quantitative real-time polymerase chain reaction
TUNEL: TdT-mediated dUTP nick end labeling
NG: Normal glucose
DAPI: 4′, 6-Diamidino-2-phenylindole
LSCM: Laser scanning confocal microscope
GAPDH: Glyceraldehyde-3-Phosphate Dehydrogenase
