# Supplementary material for: PINK1 mediated mitophagy attenuates early apoptosis of gingival epithelial cells induced by high glucose
Source: BMC Oral Health. 2022 Apr 26;22:144. doi: 10.1186/s12903-022-02167-5 (PMC9044577; doi:10.1186/s12903-022-02167-5)
Supplement: Supplementary file 1 — Additional file 1. The original picture of western blots of Figs. 1b, 1d, 2c, 3c, 4a, 4d were shown in Figs. S1b, S1d, S2c, S3c, S4a, S4d. [file 12903_2022_2167_MOESM1_ESM.docx]

The original picture of western blots was added as follows:
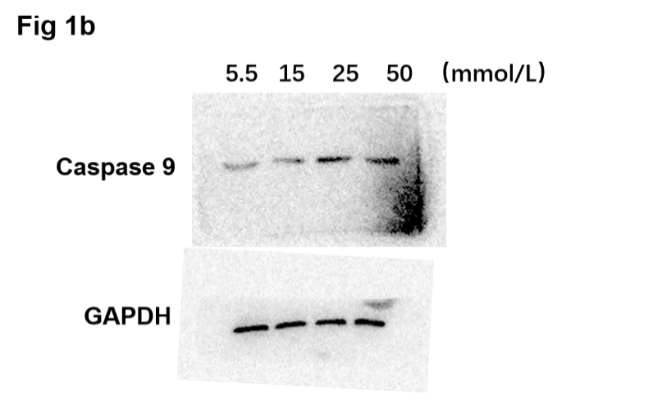


Figure S1b: The original picture of caspase 9 was detected by western blotting.


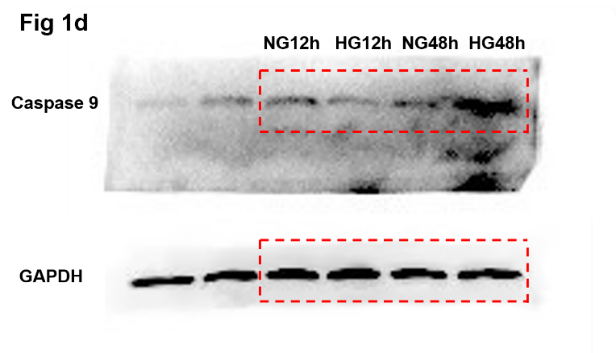


Figure S1d: The original picture of caspase 9 was detected by western blotting (the red box indicated the representative picture).


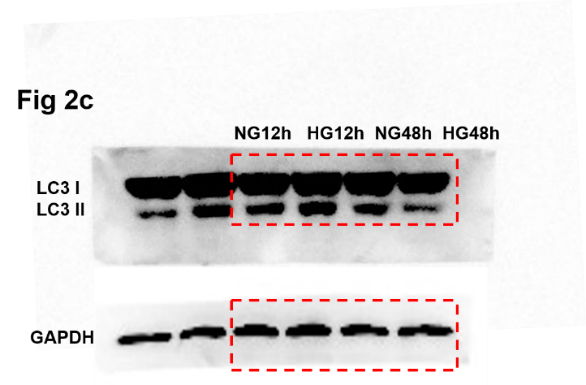


Figure S2c: The original picture of LC3 II was detected by western blotting (the red box indicated the representative picture).


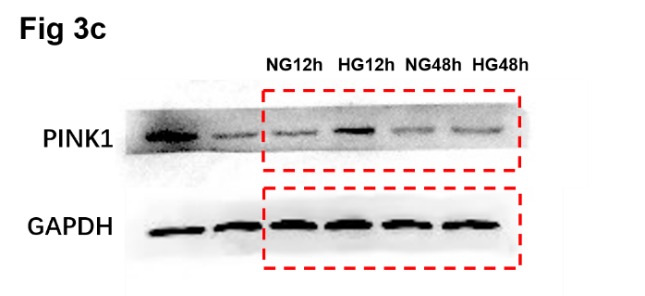


Figure S3c: The original picture of PINK1 was detected by western blotting (the red box indicated the representative picture).


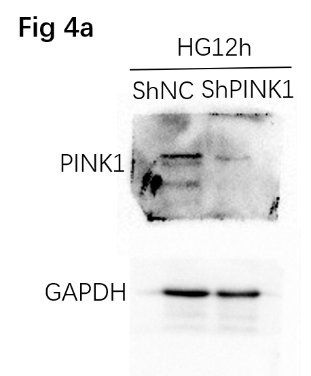


Figure S4a: The original picture of PINK1 was detected by western blotting.


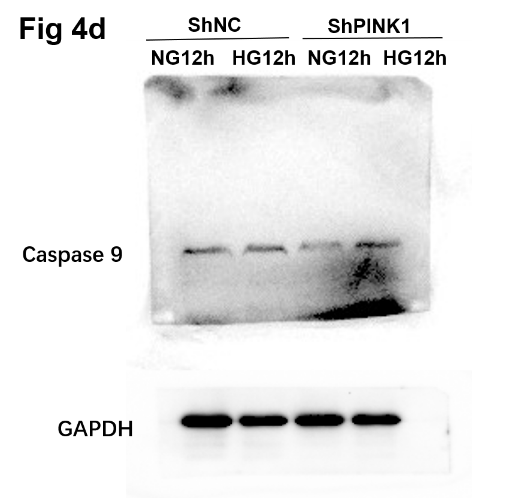


Figure S4d: The original picture of caspase 9 was detected by western blotting.
